# Crosstalk between skeletal muscle and the brain during physical activity - in search of epigenetic mechanisms

**DOI:** 10.1080/15592294.2025.2590237

**Published:** 2025-11-24

**Authors:** Cayla Boycott, Ewa Kilanczyk, Huiying A. Zhang, Jiaxi Zhang, Arian Abolhassani, Malgorzata Kubiak, Jan Celichowski, Katarzyna Kryściak, Dominika Gruszka, Joanna H. Sliwowska, Barbara Stefanska

**Affiliations:** a Food, Nutrition and Health Program, Faculty of Land and Food Systems, The University of British Columbia, Vancouver, BC, Canada; b Laboratory of Neurobiology, Department of Zoology, Faculty of Veterinary Medicine and Animal Sciences, Poznan University of Life Sciences, Poznan, Poland; c Center of Cellular Immunotherapies, Warsaw University of Life Sciences, Warsaw, Poland; d Department of Neurobiology, Faculty of Health Sciences, Poznan University of Physical Education, Poznan, Poland

**Keywords:** Muscle-brain crosstalk, physical activity, myokines, epigenetics, epitranscriptomics

## Abstract

Recent research highlights the crucial role of muscle-brain crosstalk in metabolic regulation, particularly in individuals with type 2 diabetes and obesity. Myokines, protein hormones secreted by skeletal muscle, play a crucial role in this communication, influencing brain functions such as neuroplasticity, memory, and mood. Specific myokines like cathepsin B, FNDC5/irisin and interleukin-6 have been identified as key players in this muscle-brain axis. Physical activity modulates the production of these molecular factors, enhancing muscle-brain crosstalk and influencing cellular interactions. Moreover, exercise training may lead to adaptive long-term changes in gene expression, mediated by epigenetic regulators. Metabolic pathways activated during exercise can directly impact epigenetic marks by modulating the availability of metabolic intermediates required for these modifications. In the present review, we summarize the latest findings on the association between obesity/diabetes and cognitive impairment due to hippocampal dysfunction, and elaborate on how exercise influences cognitive functions via the communication between skeletal muscle and the brain. We focus on the underlying mechanisms responsible for the muscle-brain crosstalk, emphasizing dynamic changes in the epigenome and epitranscriptome, which sheds light on novel preventive and therapeutic approaches to combat obesity and cognitive decline.

## Introduction

Obesity and type 2 diabetes (T2D) are strongly associated with cognitive decline and increased risk of dementia [1]. T2D, especially with longer disease duration, is associated with accelerated cognitive decline during aging [2]. Several mechanisms underlying obesity/T2D often overlap with features of cognitive decline such as Alzheimer’s disease (AD), including metabolic, inflammatory, vascular, and oxidative changes, suggesting a bidirectional relationship between the diseases [3,4]. In this regard, physical activity has been linked to prevention of chronic disease by strengthening the immune system, counteracting inflammation, regulating metabolism, and delaying cellular aging [5–8]. In particular, physical activity plays a crucial role in preventing T2D and improving brain health. Regular exercise has been shown to improve insulin sensitivity and glycemic control through adaptations in various organs, including adipose tissue, skeletal muscle, liver, and pancreas [9]. Indeed, increased physical activity impacts the neuromuscular system, with resistance training linked to systemic anti-inflammatory effects. Recent meta-analyses on the effect of repeated short-term and high-intensity exercise with a progressive external load (i.e., strength or resistance training) demonstrate a profound and consistent decrease in inflammatory markers in adults, including C-reactive protein (CRP), tumor-necrosis factor alpha (TNF-alpha), and interleukin 6 (IL-6) [10,11]. Adherence to muscle-strengthening exercise was also linked to reduction in obesity parameters, such as BMI and waist circumference, along with decreased inflammation during a 17-year follow up [12]. Interestingly, resistance training was shown to change the motor unit contractile properties without altering the distribution of different types of motor units in the muscle [13,14]. Improvement in contractile properties is indeed associated with increased insulin sensitivity and glucose uptake by the muscle, which can contribute to reduced low-grade inflammation and to overall health benefits.

**Figure 1.**
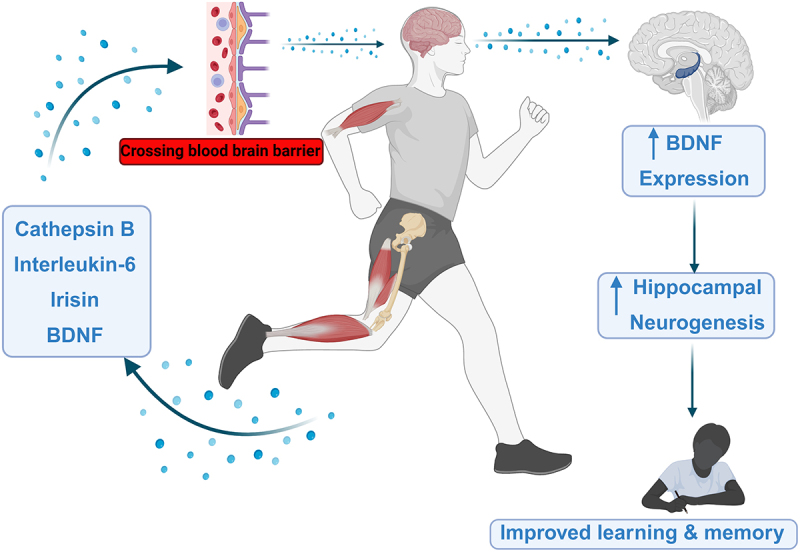
The impact of exercise on cognitive functions via BDNF- and myokine-dependent action. Physical activity leads to the release of myokines, including irisin, cathepsin beta (CTSB), interleukin 6 (IL-6), and, as recently reported, the brain-derived neurotrophic factor (BDNF) in the skeletal muscle. Those molecules travel to the brain, cross the blood-brain barrier, which is accompanied by an increase in BDNF levels in the brain, stimulation of hippocampal neurogenesis and enhancement in hippocampal-dependent learning and memory. Created in BioRender (2025) https://BioRender.com/9hulqsg.

Apart from physical performance and metabolic pathways, physical activity influences cognitive functions [15,16], resembling effects of healthy dietary patterns [6]. Indeed, regular exercise is associated with reduced cognitive decline, lower risk of all-cause dementia and AD, in particular [17]. Furthermore, structural and functional neuroimaging studies reveal that physical activity is linked to larger brain volumes in regions vulnerable to dementia, such as the hippocampus and frontal areas, as well as enhanced task-relevant activity during executive function and memory tasks [18]. These findings imply a dynamic crosstalk between the muscle and the brain and suggest that physical exercise is a promising non-pharmaceutical intervention for preventing age-related cognitive decline and neurodegenerative diseases [19,20] (Figure 1). Additionally, beyond the immediate effects of exercise, emerging research suggests that exercise induces epigenetic changes in both skeletal muscle and brain, which may influence long-term brain function and behavior across generations [21,22]. As dietary and behavioral factors have been reported to mediate modifications in the epigenome and subsequently gene expression [23,24], epigenetic mechanisms may play a role in communication between the skeletal muscle and the brain. Epigenetics, the study of heritable and potentially reversible changes in gene expression that do not involve alterations in the DNA sequence, might provide a framework for understanding the broad effects of exercise and potentially elucidating causal mechanisms that remain incompletely defined [24]. Epigenetic components, such as DNA methylation, histone modifications, chromatin remodeling complexes, and non-coding RNA mechanisms, serve as dynamic regulators of the genome, responding to environmental stimuli, such as diet, stress, and exercise, and facilitating adaptive changes [24]. Moreover, recent decades of research demonstrate that epigenetic modifications also occur on RNA, which represents a new field of epitranscriptomics [25,26]. This review will highlight the intricate interplay between the muscle and brain during physical activity and how it is mitigated in the context of T2D and obesity [27].

## Muscle-brain crosstalk during physical activity in a healthy state

The skeletal muscle during exercise acts as an active endocrine organ secreting numerous myokines that, apart from facilitating the communication within the muscle itself, travel throughout the body and regulate the function of distant organs and tissues, including brain, adipose tissue, bone, liver, gut, pancreas, and skin [19]. Indeed, exercise has been proven to be highly effective not only in maintaining muscle mass and reducing adiposity but also in improving cognitive health [28]. Exercise was shown to enhance muscle-derived myokine release, which are signaling proteins produced and released by muscle cells that are associated with increased synaptic plasticity and hippocampal neurogenesis, and thus improved cognitive functions via the muscle – brain crosstalk [29]. Evidence from both clinical [30,31] and animal studies [32,33] demonstrates that skeletal muscle dysfunction may be a key factor contributing to cognitive impairment.

Myokines, such as cathepsin B (CTSB), irisin, and IL-6, cross the brain-blood barrier (BBB), serving as messengers for communication between the skeletal muscle and the brain. The release of these messengers has been associated with an increase of brain-derived neurotrophic factor (BDNF) level in the brain [20]. BDNF is a crucial neural signal factor, essential for brain development. Interestingly, although *BDNF* is highly expressed in the nervous system, recent studies show that the skeletal muscle can also release this factor, which supports the role of BDNF as a myokine [34]. A significant body of evidence supports CTSB as an exercise-induced myokine that promotes *BDNF* expression in the hippocampus and stimulates neurogenesis [35]. For instance, running increased muscular expression of the *CTSB* gene in mice and elevated plasma levels of CTSB in both mice and humans [35,36]. Moreover, *CTSB* knockout mice were resistant to the effects of voluntary exercise, such as improved cognition and hippocampal growth [36]. However, the direct evidence for CTSB as a mediator of enhanced cognitive functions in response to exercise training in humans is missing.

Another myokine, irisin, is produced during muscular contraction in response to exercise when its precursor protein, FNDC5, is proteolytically cleaved to irisin, which is then secreted to the peripheral circulation, as evidenced by an immediate increase in serum irisin levels following exercise [37]. Irisin acts through the peroxisome proliferator-activated receptor gamma coactivator 1α (PGC-1α) [38]. These potent molecular targets of irisin mediate its broad effects on the induction of brown adipocyte-like control of energy metabolism, promotion of liver glycogen synthesis and inhibition of liver gluconeogenesis to maintain glucose homeostasis, improvement in cognition, learning, and memory, and reduction of cancer and cardiovascular disease risk [39,40]. Brain-specific effects of irisin result from its ability to cross the BBB in humans. The increased level of this myokine in the blood, achieved through adenoviral delivery of FNDC5, has been accompanied by an increase in expression of *BDNF* and other neuroprotective factors in the hippocampus, which could partially explain the improved cognitive function after exercise [41]. In addition, irisin knockdown in mouse embryonic stem cells during neural differentiation and post-neural progenitor formation significantly impeded neuronal and astrocyte maturation [42]. Moreover, brain modulatory effects and potential implications in the muscle-brain crosstalk have also been demonstrated for IL-6 [43]. Exercise-induced elevation of IL-6 is associated with beneficial effects on metabolism, insulin sensitivity, glucose uptake by the muscle, and myogenesis [44]. By crossing BBB, IL-6 May impact the brain function. Indeed, treatment of hypothalamic neuroprogenitor cells with IL-6 showed the induction of expression of genes related to neurogenesis [45].

## Muscle-brain crosstalk during physical activity in obesity and T2D

T2D and its complications are among leading causes of death globally [46]. T2D development is closely associated with obesity-induced inflammation, which impairs insulin sensitivity by damaging pancreatic beta cells, ultimately leading to insulin resistance [47]. Unhealthy diet, containing high levels of trans fats, sodium, meat, and sugary beverages, along with insufficient physical activity are major factors contributing to the epidemic of obesity and obesity-associated T2D. Regular physical activity plays a crucial role in both the prevention and management of obesity and T2D [48,49], and, as recently emphasized, in maintaining/improving mental health and cognitive functions [15,16].

Exercise has been shown to influence the hippocampus more profoundly than any other part of the brain. The hippocampus, as a highly plastic brain structure, is capable of substantial reorganization in response to environmental factors, including diet and physical activity, and generation of new neurons in adulthood through a process known as adult neurogenesis [50,51]. Of importance, obesity measured by higher visceral adipose tissue was associated with lower hippocampal volume [52]. Patients with BMI over 30 exhibited hippocampal atrophy compared to individuals with normal weight [53]. Even healthy individuals without T2D, whose blood glucose levels were in the upper normal range, had a detectable reduction in the hippocampal volume [54]. In preliminary investigation on the effects of Western-type diet, an increased fat consumption was significantly related to decreased left hippocampal volume in children [55]. Importantly, studies in rodents and humans have demonstrated that exercise is connected to the increased hippocampal volume and blood flow to this part of the brain, promoting neurogenesis and synaptic plasticity [56].

### Physical activity is associated with improved cognitive functions via BDNF in obesity and T2D

BDNF appears to play a dominant role in mediating the effects of exercise on the hippocampus, partly due to the release of myokines [57]. Rodent studies have demonstrated increased *Bdnf* mRNA and Bdnf protein expression within the hippocampus in response to wheel running [58]. There is also strong mechanistic evidence supporting BDNF-mediated effects of exercise on improved cognitive functions, such as memory and learning in animals [59]. Importantly, these findings are supported by studies in humans [60]. By increasing BDNF levels, physical activity is believed to have protective effects against Alzheimer’s disease (AD) [61]. However, obesity and T2D often exhibit lower baseline levels of BDNF, which are linked to cognitive deficits and mood disorders [62–64]. Obesity is associated with chronic low-grade inflammation and metabolic dysregulation, which can impair myokine signaling pathways and reduce BDNF levels [64]. Indeed, obesity has been shown to be associated with cognitive decline and increased risk of AD and other neurodegenerative disease in a cohort study [65]. A recent meta-analysis has revealed that while acute exercise was linked to significantly increased BDNF levels in individuals with obesity, regular long-term exercise did not show a significant increase in BDNF levels. Yet, others have found that BDNF levels rise with aerobic exercise along with improvements in mood in individuals with obesity [64,66]. These findings suggest that while acute bouts of exercise lead to transiently elevated BDNF levels in individuals with obesity, the long-term effects of regular exercise on BDNF may be influenced by factors such as exercise type, intensity, duration, and individual metabolic health, among others. Importantly, decrease in adiposity upon exercise may further mediate BDNF effects on neurogenesis and improved cognition. Therefore, personalized exercise interventions may be necessary to optimize BDNF-mediated cognitive benefits in populations with obesity and T2D.

### The action of irisin in obesity and T2D

Alterations in the muscle-brain crosstalk observed in metabolic disorders such as obesity and T2D may be driven primarily by changes in myokine levels. Interestingly, most studies have reported that irisin is positively associated with BMI and markers of insulin resistance. A meta-analysis involving 1,005 obese patients and 1,242 control subjects revealed that individuals with obesity had higher circulating irisin levels [67]. Thus, high levels of irisin may represent a physiological response and ‘hyperirisinemia’ may serve as a compensatory mechanism to counteract observed irisin resistance and maximize irisin’s anti-obesity and anti-hyperglycemic effects [39,40]. Indeed, irisin levels are inversely correlated with age and the duration of disease, with low levels detectable in patients with pre-diabetes and T2D [68]. High-irisin individuals with obesity have a reduced risk of comorbidities, such as T2D [69]. Those patients also have a better prognosis, improved lipid metabolism, and a lower probability of insulin resistance-related complications [70].

Similar to human studies [71], animal models of obesity and T2D have confirmed the role of irisin in metabolic regulation [72]. Experiments on mice fed a HFD and injected with FNDC5-expressing adenoviral particles or recombinant human irisin showed lower glucose levels after intraperitoneal glucose infusion and lower fasting insulin levels, suggesting that irisin can reduce insulin resistance [73]. Similarly, diabetic HFD-fed mice treated with irisin showed improved glucose tolerance, insulin sensitivity and blood lipid profile, and/or reduced fat mass [74]. Thus, supplementation with recombinant irisin or potentially exercise-activated irisin might be a successful strategy to combat obesity.

Moreover, increasing evidence indicates that the browning of WAT protects against obesity and obesity-related metabolic disease [71]. While WAT is linked to energy storage and release of inflammatory cytokines, BAT is metabolically active and implicated in thermoregulation. Indeed, browning of adipose tissue reduces fasting insulin levels, increases glucose tolerance, and decreases body weight [75]. Of particular interest, exercise-induced release of irisin has been demonstrated to contribute to browning of white adipocytes [73]. Exposure to irisin in vitro and in vivo led to the induction of a program of brown fat-like development [73]. Indeed, resistance training program increases plasma irisin levels in older adults and leads to a reduction of visceral adipose tissue [76]. Animal studies in aged mice [77] and obese rats [78] show a similar effect of resistance training program, with increased irisin levels in the skeletal muscle and serum. Elevated irisin in both skeletal muscle and serum was also reported after a single treadmill trial in obese animals [79,80].

Importantly, irisin is believed to be a major contributor to exercise-induced cognitive improvements. While physical activity increases irisin levels, neurological disease is linked to low irisin in plasma [81]. Of importance, the increase of circulating irisin has been shown to be sufficient in alleviating both the cognitive deficit and neuropathology in AD mouse models [82].

### The action of CTSB in obesity and T2D

Another important myokine, CTSB, plays a crucial role in metabolic regulation in obesity and T2D, however CTSB-mediated effects appear to be in contrast with irisin action and actually exacerbate inflammation [83]. CTSB levels are higher in obese adipose tissue, as compared to adipose tissue in lean animals, and are linked to lipolysis and liposomal dysfunction [84]. During hypertrophy of WAT in obesity, CTSB is released into the cytosol, leading to autophagosome accumulation in WAT. It causes adipocyte cell death and at the same time promotes infiltration of macrophages, resulting in low-grade inflammation characteristic of metabolic syndrome. Increased autophagosome formation in WAT and higher levels of CTSB were found in mice fed HFD. Importantly, while exercise training enhanced autophagosome formation in WAT, it reduced *CTSB* expression, potentially lowering the inflammatory state [85].

On the other hand, CTSB has been shown to have a neuroprotective role in other studies [86,87]. In a CTSB-deficient mouse model of AD with overexpression of human amyloid protein precursors (hAPP), increased amyloid deposition was observed in the hippocampus and cortex (the exact part not specified). Lentiviral overexpression of CTSB, in turn, reduced pre-existing amyloid deposits in aged-hAPP mice [87]. It is further illustrated that adenoviral overexpression of CTSB in a transgenic mouse model of AD leads to amelioration of AD-like pathologies by reducing hippocampal amyloid depositions and promoting learning and memory [86]. Hence, increasing CTSB levels are expected to be a promising target for AD intervention. However, given the negative impacts of CTSB in the context of obesity and neuroprotective role of CTSB induced by exercise, future studies investigating the impact of exercise-induced release of CTSB in a model of obesity-associated AD will be of great importance.

### The action of IL-6 in obesity and T2D

IL-6 is a complex cytokine with both pro- and anti-inflammatory properties. Its signaling occurs through two main pathways: classical and trans-signaling, each with distinct effects [88]. Classic signaling involves IL-6 binding to the membrane-bound IL-6 receptor (IL-6 R), leading to anti-inflammatory and regenerative responses, while trans-signaling involves IL-6 binding to the soluble IL-6 R (sIL-6 R), which then initiates pro-inflammatory responses [88]. IL-6 trans-signaling is implicated in recruiting macrophages to adipose tissue in obesity, contributing to inflammation [89]. Indeed, in individuals with obesity and T2D, chronic low-grade inflammation, characterized by elevated IL-6 levels in various tissues, can contribute to reduced insulin sensitivity and impaired glucose metabolism [90]. These systemic effects from obesity-related increase of IL-6 trans-signaling are also thought to play a role in chronic neuroinflammation and the progression of AD [91]. On the other hand, classic IL-6 is postulated to have a protective role in the brain by activating Signal Transducer and Activator of Transcription 3 (STAT3), a key transcription factor in neurons, playing a role in neural development and neuronal survival [92,93]. Notably, acute bouts of exercise have been shown to increase IL-6 signaling in the brain along with increased activity of STAT3 and reduction of key enzymes in amyloidogenic cascade in the prefrontal cortex and hippocampus of the brain, indicating the beneficial role of IL-6 in the context of AD [94]. However, whether these transient spikes of IL-6 are enough to counteract obesity- or T2D-associated chronic inflammatory signals remain elusive.

## Epigenetics and epitranscriptomics

**Figure 2.**
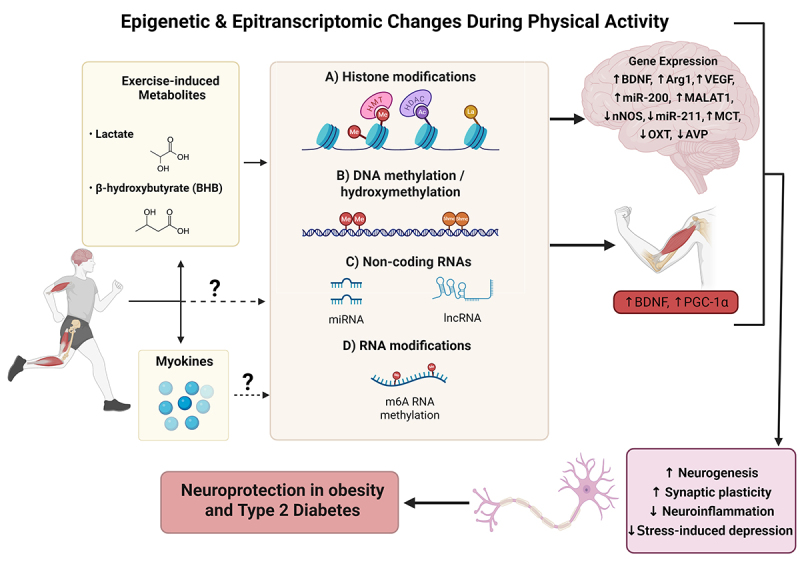
Epigenetic and epitranscriptomic changes during physical activity. Exercise-induced metabolites, such as lactate and beta-hydroxybutyrate (BHB), cross the blood-brain barrier and impact the epigenetic and epitranscriptomic modifications at gene-specific loci. These changes in epigenetics and epitranscriptomics are accompanied by remodeling of expression of key genes involved in increased neurogenesis and synaptic plasticity, and decreased neuroinflammation and stress-induced depression. The exercise-induced metabolites also impact epigenetic regulation of BDNF and PGC-1alpha in the skeletal muscle. Myokines and other factors induced by exercise may also induce epigenetic remodeling in the brain and muscle via mechanisms that are yet to be explored. Overall, epigenetic and epitranscriptomic remodeling might be implicated in neuroprotective effects of exercise in obesity and type 2 diabetes (T2D). Created in BioRender (2025) https://BioRender.com/6bbmqbm.

In recent decades, research has indicated the role of various epigenetic and epitranscriptomic mechanisms in the regulation of the muscle-brain crosstalk upon exercise in a healthy and obesogenic state (Figure 2). Epigenetics refers to the study of changes in gene expression that do not involve alterations to the underlying DNA sequence [95]. These changes are often mediated by chemical modifications to DNA or histone proteins, such as DNA methylation and histone acetylation, which can regulate gene activity by turning genes on or off.

DNA methylation typically suppresses gene activity by adding methyl groups to cytosine bases, while histone modifications, including acetylation and methylation, can either condense or relax chromatin structure, thereby repressing or activating gene transcription, respectively [96–98]. Non-coding RNAs (ncRNAs), such as microRNAs and long non-coding RNAs, further modulate gene expression by interacting with mRNA or chromatin-modifying complexes, influencing processes like mRNA degradation and chromatin remodeling [99]. Finally, epitranscriptomics, the study of chemical modifications on RNA molecules, adds another layer of gene regulation. A prominent example is N6-methyladenosine (m6A), a modification that affects RNA stability, splicing, and translation, thereby influencing protein synthesis and cellular function [100].

Collectively, these mechanisms are dynamic and responsive to environmental factors like physical activity, playing crucial roles in muscle adaptation and brain function. However, in the context of obesity, these regulatory processes may be disrupted, potentially impairing the beneficial effects of exercise on muscle-brain communication.

## Epigenetic and epitranscriptomic changes in the skeletal muscle during physical activity

Physical activity may lead to stable and lasting epigenetic modifications that can have enduring effects on gene expression and muscle function. For instance, a study by Lindholm et al. demonstrated that three months of endurance training resulted in significant genome-wide changes in DNA methylation in human skeletal muscle [101]. These alterations were predominantly found in enhancer regions and were associated with the regulation of genes involved in muscle structure and metabolism. Importantly, these methylation changes correlated with modifications in gene expression, suggesting that long-term exercise can reprogram the epigenetic landscape of skeletal muscle to support enhanced function and adaptation. Furthermore, a recent study on endurance-trained individuals revealed distinct DNA methylation patterns in skeletal muscle compared to untrained individuals, particularly in genes related to muscle fiber type and transcriptional regulation [102]. These epigenetic modifications were associated with altered gene expression responses to acute exercise, suggesting that long-term training induces stable epigenetic changes that prime skeletal muscle for enhanced adaptability.

This concept of ‘epigenetic memory’ in skeletal muscle, where prior exercise induces lasting DNA methylation changes, raises intriguing questions about its potential influence on muscle-brain crosstalk. While direct evidence linking muscle epigenetic memory to alterations in brain function is still emerging, several mechanisms suggest a plausible connection.

It is yet to be fully elucidated whether exercise-induced epigenetic modifications in skeletal muscle may lead to sustained changes in the expression of myokines such as irisin and IL-6 that are known to cross the BBB and influence brain function. If epigenetic memory enhances the baseline expression or responsiveness of these myokines to subsequent exercise, it could potentiate their neuromodulatory effects. While the precise pathways remain to be fully elucidated, these findings collectively imply that the epigenetic adaptations in muscle resulting from regular exercise could have downstream effects on brain health and function. Further research is needed to delineate these mechanisms and their implications for interventions targeting cognitive and neuromuscular health.

## Epigenetic changes in the brain in response to physical activity

### Physical activity leads to remodeling of the DNA methylation pattern in the brain

Exercise-induced epigenetic changes are not confined to the skeletal muscle but also significantly impact the brain. For example, studies have shown that physical activity leads to changes in DNA methylation patterns and DNA methyltransferase (DNMT) expression in the hippocampus, a region critical for learning and memory [103]. These changes occur globally and at specific gene promoters. A study investigating a short-term running exercise in mice demonstrated that exercising animals have the increased DNA methylation at the *Nos1* promoter in the ventral hippocampus, which is accompanied with reduced expression of neuronal nitric oxide synthase (*nNOS*), a gene associated with anxiety-like behaviors [104]. On the other hand, DNA methylation at the *Bdnf* promoter was reduced, which coincided with enhanced *Bdnf* expression.

In addition to DNA methylation, Jessop and Toledo-Rodriguez [104] explored the effects of voluntary exercise on Ten-Eleven Translocation (TET) enzymes, which mediate DNA hydroxymethylation, 5hmC mark. They found that exercise is linked to restored age-related declines in *TET1* and *TET2* expression in the hippocampus and increased 5hmC levels at the *miR-137* promoter, a miRNA involved in adult neurogenesis. These molecular changes correlated with improved performance in memory tests.

### Physical activity impacts histone modifications in the brain

Histone modifications are equally important in mediating the exercise-induced brain adaptation. Notably, it was found that exercise is associated with reduced anxiety symptoms and concomitant enhancement of active and reduction of repressive histone marks at genes related to anxiety repression. For example, an exercise intervention resulted in increased total *Bdnf* mRNA levels, along with enhanced histone H3 acetylation (H3Ac), a mark of active transcription, at its promoter. These changes were associated with decreased expression of histone deacetylase enzymes [105]. Similarly, another study showed that chronic stress leads to reduction of the histone methyltransferase *G9a* and to demethylation of histone H3 lysine 9 (H3K9me2) at oxytocin (*OXT*) and arginine vasopressin (*AVP*) promoters in the basolateral amygdala in the brain, which may, at least partially, explain upregulation of these genes and depressive behaviors. Remarkably, these aberrant changes were mitigated in response to exercise, demonstrating therapeutic potential through epigenetic reprogramming [106].

### Physical activity impacts non-coding RNA mechanisms and epitranscriptomics in the brain

In recent years, growing evidence has highlighted the critical role of non-coding RNA-mediated mechanisms in regulating exercise-induced brain plasticity, suggesting that these molecules contribute to neural adaptation and cognitive enhancement associated with physical activity. A recent study investigated exercise-responsive miRNAs in the brain using high-intensity intermittent swimming training (HIST) in rats [107]. Through deep sequencing and bioinformatics analysis, novel miRNAs have been identified that are differentially expressed in response to HIST. Among them, miRNAs of the *miR-200* family were strongly upregulated, suggesting their central role in the exercise-induced brain adaptation. Target prediction and validation using qRT-PCR and immunohistochemistry revealed that these miRNAs regulate key genes, such as *BDNF*, *IGF-1*, *NGF*, and *c-Fos*, which are involved in neurogenesis, synaptic plasticity, and neuronal survival.

Similarly, Yan et al. [108] demonstrated that chronic restraint stress in mice led to decreased m6A levels in the brain, correlating with anxiety-like behaviors. Upon exercise, disrupted m6A levels were restored, which was associated with increased expression of methyltransferases *METTL3* and *METTL14* and reduced expression of RNA demethylase *ALKBH5*. These changes were accompanied with enhanced cortical activity and improved stress resilience. Strikingly, an exercise intervention also led to increased one-carbon metabolism in the liver and production of SAM, the ubiquitous methyl donor. Mechanistic studies confirmed that hepatic SAM biosynthesis is required for exercise-mediated attenuation of anxiety-like behaviors. These findings highlight the liver-brain axis linking exercise to epigenetic regulation and providing new insights into brain functions.

## Epigenetics and epitranscriptomics in the crosstalk between the skeletal muscle and the brain

The interplay between the skeletal muscle and the brain during exercise has garnered significant attention, with growing evidence suggesting that epigenetic mechanisms may play a crucial role. Exercise-induced metabolites, such as β-hydroxybutyrate (BHB) and lactate, are increasingly recognized for their ability to influence gene expression and modulate the activity of epigenetic enzymes across the body, particularly in the brain [109] (Figure 2).

One study investigated how BHB, a ketone body released by the liver and utilized as an energy source in the brain under low-glucose conditions, impact the muscle and brain functions [109]. In skeletal muscle cells treated with BHB, cell viability improved and BDNF expression increased. Animal studies with aged mice demonstrated that endurance exercise leads to increased BHB concentrations in the serum and skeletal muscle, correlating positively with muscle strength and cognitive functions. Since BHB acts as an inhibitor of HDACs, it potentially increases BDNF by enhancing histone acetylation and promoting chromatin relaxation [110].

Similarly, lactate, a metabolite produced during exercise, which demonstrated potential antidepressant and resilience-promoting effects in a mouse model of chronic social defeat stress, impacts the brain functions [111]. Male mice subjected to stress sessions showed improved anxiety symptoms following lactate administration. Molecular analyses revealed that a stress-induced reduction in hippocampal HDAC2 and HDAC3 levels was reversed in response to lactate, which could be critical for regulating gene expression and promoting stress resilience. Furthermore, lactate exhibited antidepressant effects, even after the onset of depression, along with increased histone acetylation and decreased HDAC5 levels. Although the study did not directly link these effects to exercise, the findings suggest that lactate may mimic the beneficial impacts of exercise on brain function through HDAC-dependent pathways.

The communication between exercising muscle and the brain may also involve changes in the kynurenine pathway via PGC-1α. Exercise has been shown to reduce DNA methylation and to upregulate *PGC-1α* in human skeletal muscle biopsies [112]. Notably, subsequent research linked *PGC-1α* upregulation to kynurenine metabolism, highlighting a novel pathway through which exercise may protect against stress-induced depression [113]. PGC-1α inhibits the stress-activated kynurenine pathway of tryptophan degradation, facilitating the conversion of kynurenine into kynurenic acid. The latter cannot cross the blood-brain barrier and thus kynurenine detrimental impact on the brain is halted. Together, these studies reveal how exercise-driven changes in DNA methylation influence not merely muscle physiology, but may also play a role in the muscle-brain crosstalk.

### Epigenetic and epitranscriptomic alterations affecting neural health in the context of obesity and T2D

Obesity and T2D are not merely metabolic disorders but also conditions characterized by significant and often persistent epigenetic dysregulation that can impact neurological health. For instance, aberrant DNA methylation is implicated in both obesity and diabetes-related cognitive decline. For instance, a study in mice found that a HFD and streptozotocin (STZ)-induced diabetes leads to altered DNA methylation patterns in the hippocampus and global hypermethylation within the neurons [114]. This was accompanied by an increase in DNA methylating enzymes, DNMTs, and decreased in molecular chaperones and synaptic proteins. Importantly, treatment with a DNMTs inhibitor, 5-aza-2’-deoxycytidine, partially reversed these negative effects, improving both neuroanatomical and behavioral outcomes. Moreover, in pre-diabetic individuals with insulin resistance, DNA hypermethylation at brain *CPT1A*, the carnitine palmitoyltransferase essential for mitochondrial β-oxidation of long-chain fatty acids, was found to be significantly associated with a higher risk of cognitive impairment and AD-related indices [115].

Furthermore, the evidence from an experimental mouse model suggests that diet-induced obesity can lead to epigenetic dysregulation of the dopamine system, and to DNA methylation and silencing of dopamine-related genes in the central nervous system [116]. Suppressed dopamine release, in turn, leads to the activation of inflammasome implicated in neuroinflammation and neurodegenerative disease [116]. Interestingly, in a murine model, a history of diet-induced obesity was shown to lead to long-lasting changes in the innate immune system, specifically in adipose tissue macrophages, that were not reversed after dietary weight loss, indicating a persistent ‘memory’ of past obesity in these cells [117].

Progression from HFD-mediated obesity to STZ-induced diabetes in mice demonstrated epigenetic dysregulation of the neuroprotective protein BDNF [118]. Reduced expression of *Bdnf* was found in the prefrontal cortex along with cognitive impairment. This was associated with hypermethylation of the *Bdnf* promoter and increased expression of DNA methyltransferases, *Dnmt1*, *Dnmt3a*, and *Dnmt3b* [118].

Active DNA demethylation mediated by TET enzymes has also been identified as a crucial player in the pathological mechanisms of diabetic cognitive dysfunction (DCD), such as diabetes-induced neuronal apoptosis [119]. In the HFD-induced diabetic mice model, significantly lower levels of TET2 protein and 5hmc, accompanied by a higher proportion of apoptotic cells, were observed in the cerebral cortex, compared to healthy control mice. Expression of pro-apoptotic proteins, such as *Bax* and *Bak*, was found significantly higher whereas anti-apoptotic proteins were downregulated in the diabetic group.

Apart from DNA methylation, histone modifications also play a crucial role in diabetes-associated neural dysfunction. A recent study demonstrated that diabetic mice exhibit a significant reduction in H3K9/14 and H4K12 acetylation in the cortex (the exact part not specified) and hippocampus, accompanied by elevated HDAC activity [120]. Histone hypoacetylation was linked to the transcriptional repression of memory-related genes such as *BDNF*, as well as synaptic markers *SYP* and *PSD-95*, which are critical for synaptic plasticity and cognitive functions. Indeed, pharmacological inhibition of HDACs was shown to increase H3/H4 acetylation and expression of *BDNF*, *SYP*, and *PSD-95*, and alleviate cognitive deficits associated with T2D.

Dysregulation of miRNAs has also been implicated in obesity- or T2D-associated cognitive decline and neurodegeneration. For example, downregulated expression of *miR-146a-5p* was found in the hippocampus of diabetic rats fed high-fat or high-sucrose diet [121]. This change was accompanied by increased hippocampal neuron cell apoptosis, and upregulated expression of thioredoxin-interacting protein (*TXNIP*), interleukin *IL-1β* and tumor necrosis factor *TNF-α*, suggesting diabetes-induced endoplasmic reticulum stress and neuroinflammation. In the high glucose-treated primary hippocampal neurons, depletion of *miR-146-5p* revealed its inhibitory role in regulation of *TXNIP*. Furthermore, in T2D patients with cognitive impairment, increased expression of *miR-34a-5p* and decreased expression of sirtuin 1 (*SIRT1*) in peripheral blood was reported, as compared with T2D patients without cognitive impairment and healthy controls [122]. A depletion of *miR-34a-5p* demonstrated its inhibitory role in regulation of *SIRT1* [122]. Indeed, other studies previously reported on neuroprotective effects of SIRT1 and overexpression of *miR-34a-5p* in cognitive impairment and AD-like pathology [123].

In addition to epigenetic changes, epitranscriptomic disruptions in key brain regions such as the hippocampus and hypothalamus further compound the metabolic and neurological dysfunctions associated with obesity/T2D. Using a HFD-induced diabetic cognitive impairment mouse model, Cao et al. revealed differential patterns of m6A modification in the hippocampus [124]. Genes associated with T2D and obesity had low levels of m6A and were upregulated, whereas genes associated with neurodevelopment and neurological health had high m6A and were downregulated. It could potentially contribute to neuropathological changes underlying cognitive impairment in diabetic mice.

### Effects of exercise on neuroepigenetics/neuroepitranscriptomics in the context of obesity and T2D

Emerging evidence suggests that exercise can counteract obesity or T2D-associated neuroepigenetic alterations by offering a potential strategy to mitigate neuroinflammation and enhance brain functions (Figure 1).

Exercise interventions have been demonstrated to induce beneficial changes in gene expression related to hippocampal functions in obesity and T2D via miRNA-mediated mechanisms. For instance, voluntary exercise in a diet-induced obesity mouse model has been shown to reduce *miR-211* overexpression, mitigating hypothalamic inflammation and rescuing genes involved in immune responses, synaptic modulation, and energy homeostasis. These molecular changes were accompanied by reduction in weight gain and body fat mass [125]. In an HFD-induced T2D mouse model, aerobic exercise led to the upregulation of the lncRNA MALAT1 in serum exosomes [126]. Further mechanistic studies showed that MALAT1 competitively inhibited miR-382-3p and subsequently increased *BDNF* expression in hippocampal neurons [126]. This regulation improved hippocampal neuron proliferation, reduced apoptosis, and alleviated cognitive impairment related to T2D.

Beyond the effects on microRNAs, exercise also influences epitranscriptomic modifications that regulate brain functions. Notably, the expression of *FTO*, the m6A demethylase, is modulated by both diet and exercise, with significant implications for metabolic and neural health [127]. Research indicates that *FTO* is upregulated in the brain in conditions of obesity, particularly in the hypothalamus and can be associated with leptin resistance, a condition that disrupts appetite and energy balance [128]. Furthermore, in patients with T2D, *FTO* expression at both mRNA and protein level is elevated compared to healthy controls, with more pronounced increases seen in those with severe T2D [127]. These elevated levels of FTO are also positively correlated with obesity, insulin resistance, and blood glucose indices, indicating a strong link between FTO and the pathogenesis of T2D. Of interest, long-term exercise training has been demonstrated to downregulate *Fto* expression in the hippocampus and hypothalamus of mice, which was associated with an increase in m6A levels [129]. Given that FTO is an m6A demethylase, exercise-induced reduction of FTO likely contributes to the observed increase in m6A.

Moreover, exercise may also have an impact on mitigation of obesity-induced gene expression changes across generations. Research on the offspring of obese mothers shows that exercise early in life is associated with changes in hypothalamic gene expression, particularly in appetite regulators and *FTO* [130]. These changes may counteract the adverse effects of maternal obesity on energy balance and metabolic health. By targeting critical hypothalamic pathways, early-life exercise may help reprogram the inherited epigenetic landscape, potentially reducing susceptibility to neuroinflammation, metabolic dysregulation, and related cognitive impairments later in life. Similarly, maternal exercise was also shown to prevent HFD-induced hypermethylation of the Pgc-1α gene promoter, a key protein that regulates mitochondrial biogenesis and energy metabolism, in offspring skeletal muscle, enhancing Pgc-1α expression and ameliorating age-associated metabolic dysfunction [131]. This may have implications for brain health as PGC-1α plays a crucial role in influencing neuronal function, mitochondrial health, and synaptic maintenance [132]. The exercise-induced epigenetic modifications, that are observed in offspring, may play a crucial role in the intergenerational health impact of exercise, potentially counteracting the negative effects of maternal obesity on offspring neural health.

Another key mechanism through which exercise may exert neuroprotective effects is by increasing lactate production, which serves as both an energy source and a signaling molecule that can modulate neuroepigenetic processes. Lactate can be transported into neurons, where it may be used as the substrate for histone lactylation (Kla), a newly defined histone modification, that have been shown to play a beneficial role in neuroprotection [133]. In an AD mouse model, exhibiting neuroinflammation and cognitive decline, both exercise and exogenous lactate administration reduced microglial hyperactivation and increased the number of reparative microglia [134]. Elevated lactate levels, observed in both the plasma and brain of mice following exercise training, were associated with increased histone H3 lactylation, particularly at H3K18. This lactylation promoted the expression of homeostatic and reparative genes in microglia, such as *Arg1* and *VEGF*, facilitating a shift from a damaging pro-inflammatory state to a reparative phenotype. Consequently, this mechanism alleviated neuroinflammation and enhanced cognitive performance in the animal models.

Building on the understanding of exercise-mediated epigenetic changes, particularly Kla, as a beneficial pathway in neuroprotection, research in T2D models reveals another facet of how exercise can positively impact brain function. In a diabetic rat model, which exhibited memory dysfunction and dysregulated hippocampal glycometabolism, four weeks of moderate exercise resulted in improved spatial memory and restored expression of monocarboxylate transporter 2 (*MCT2*) in the hippocampus [135]. Although this study did not directly measure histone modifications, it is important to note that MCT2 plays a role in transporting lactate into neurons, suggesting a potential link to Kla. The diabetic rats also exhibited elevated glycogen levels and decreased *MCT2* expression in the hippocampus, which was improved with exercise. This highlights the link between exercise-induced changes in glycometabolism and memory improvement, suggesting that the availability of lactate as an energy source and a signaling molecule may be a key factor.

Of particular importance, lactate levels, as well as expression of its transporters (*MCT1, MCT2, MCT4*), decrease in the context of AD, underscoring the critical role of this metabolite in maintaining neural health [136]. Interestingly, while reduced lactate availability is linked to neurodegeneration in AD, there are conflicting findings in the context of T2D. Increased lactate levels have been associated with diabetes-related cognitive decline in rats, suggesting a more complex relationship between lactate metabolism and brain health that may depend on disease-specific mechanisms or context [137].

Overall, lactate may be an important signaling link in the muscle-brain crosstalk, since it is produced by the skeletal muscle during exercise and then transported to the brain (Figure 2). Exercise may thereby counteract some of the negative metabolic effects of these disorders and restore a more beneficial balance of metabolic activity in cells.

## Conclusions and future directions

Despite the growing body of evidence demonstrating significant exercise-induced epigenetic changes, the skeletal muscle-brain crosstalk and epigenetic modifications within these organs remain poorly understood. In particular, the potential bidirectional relationship between signaling molecules (e.g., myokines, BDNF) and epigenetic patterns in the skeletal muscle and the brain require further exploration (Figure 2). While metabolites like lactate and BHB are known to influence brain and muscle epigenetics and epitranscriptomics (Figure 2), their precise signaling mechanisms remain underexplored. How these systemic signals integrate with myokines and central pathways, such as RNA methylation and histone lactylation, is still unclear. Another important aspect is controlling for different variables that can impact the cognitive functions and exercise performance (e.g., diet, sleep, mental state) in mechanistic and cause-effect investigations. Furthermore, the extent to which peripheral metabolic improvements through exercise translate to long-term neuroprotection, particularly in aging populations or advanced metabolic disease, require further investigation. Closing these gaps and identifying key molecular mechanisms of multi-organ epigenetic remodeling will provide a more comprehensive understanding of muscle-brain communication, offering novel insights into exercise’s therapeutic potential for combating metabolic and neurodegenerative diseases.

## Data Availability

As this is a review article, no original research is presented and data availability statement is not applicable.
